# Health economic evaluation of a medication safety intervention in elderly care: identifying causal effects in a multi-center quasi-experimental study design

**DOI:** 10.1186/s12913-025-12898-0

**Published:** 2025-05-30

**Authors:** Benedikt Langenberger, Martin Siegel, Reinhard Busse, Verena Vogt

**Affiliations:** 1 Technical University of Berlin, Berlin, Germany; 2 Hasso Plattner Institute, Potsdam, Germany; 3 Jena University Hospital, Jena, Germany

## Abstract

The high prevalence of multimorbidity in the aging population necessitates complex medication regimens, increasing the risk of adverse drug events (ADEs) and hospital admissions. This paper evaluates an intervention aimed at improving medication safety for northeastern and western Germany under real-world conditions, thereby providing a pragmatic approach to the challenges of multi-center studies with staggered intervention starts and voluntary participation. The analysis utilizes iterative Propensity Score Matching (PSM) followed by a Difference-in-Differences (DiD) estimator to navigate the methodological complexities and assess the intervention’s effectiveness and cost-effectiveness. Results reveal a significant reduction in ADE-related hospital admissions by 27.5% and overall hospital admissions by 17.5%. We find that the intervention is cost-effective at an incremental cost-effectiveness ratio (ICER) of €15,169.66 per averted ADE and €4,180.61 per averted hospital admission. This study illustrates for evaluating complex health interventions in real-world settings and underscores the importance of balancing health outcomes improvements with economic considerations in aging populations.

## Introduction

In health economic evaluations of complex interventions in real world healthcare settings, multi-center studies with voluntary participation, a staggered starting of the intervention in different centers, and subsequently a retrospective selection of controls from health insurance data, can create numerous challenges for researchers interested in the effectiveness of such interventions. This paper takes the evaluation of an intervention to improve the safety of medication for care-dependent elderly people living in nursing homes or receiving ambulatory nursing care (home care) as an example and presents a strategy to evaluate such an intervention.

The prevalence of multimorbidity is high in aging societies, frequently resulting in polypharmacy – defined as taking five or more different medications simultaneously – and complex medication regimes among elderly patients. For instance, evidence suggests that German nursing home residents with renal failure have a polypharmacy rate of more than 50 % [1]. Unfortunately, there is a lack of nation wide studies on the rate of medications for the general nursing home population in Germany. Medications for these patients are prescribed by physicians which are usually operating from private offices and are visiting the facility. Polypharmacy is associated with higher risk for adverse drug events (ADE) [2–4], may increase rates of hospital admissions and adversely affect patients’ physiological and cognitive well-being [5]. Polypharmacy treatment with cautiously chosen drug combinations may be adequate in specific cases [6], but critical evaluations are crucial to maintain the quality and safety of the pharmacotherapy. We present the evaluation of an intervention that combined the sensitization of caregivers through specialized training sessions with the deployment of computer-assisted analyses of medication plans to identify potential drug interactions, potentially unnecessary medications, or potentially inadequate medications (PIMs), where appropriate [7]. The aim of the intervention was to reduce the incidence of ADEs (primary outcome) and hospital admissions (secondary outcome) by reducing the prevalence of polypharmacy and the incidence of PIM prescriptions.

A significant challenge in the evaluation of this intervention stemmed from the staggered initiation of the intervention across multiple treatment centers, combined with the voluntary enrollment of participating nursing facilities (both nursing homes as well as ambulatory nursing care providers) and care-dependent individuals, and the retrospective selection of controls from routine data. Such complexities may introduce biases, which may lead to less robust or even misleading results. To navigate these challenges and ensure the integrity of our findings, we employed an approach combining iterative Propensity Score Matching (PSM) with a difference-in-differences estimator (DiD) to draw causal inference. The objectives of this paper are twofold: First, we demonstrate how increased awareness and screenings of medication plans can improve the medication safety and health outcomes among a care-dependent elderly population, and second, we describe the methodological challenges encountered and elucidate the strategies employed to overcome them.

The remainder of this paper is organized as follows: The next section describes the study design and the data available for the evaluation, and defines the different measures included in the analysis. The third section describes the tailored PSM approach used to obtain a suitable control group and introduces the difference-in-difference (DiD) estimation strategy. The fourth section presents the results, which are discussed in the fifth section. We draw our conclusions in the sixth section.

## Methods

### Intervention

The primary goal of the intervention (German title of the intervention: *Optimierte Arzneimittelversorgung für pflegebedürftige geriatrische Patienten* - short version: *OAV*) was to reduce ADEs by targeting polypharmacy and potentially inappropriate medications among geriatric patients. To achieve this, experienced geriatric pharmacists provided theory-based training sessions for interdisciplinary teams-comprising nurses, physicians, and pharmacists-before the intervention began.

ADE risk identification combined ongoing clinical observation by the trained care teams with computer-assisted screening facilitated by the software VERIKO PT®. Patients flagged by these methods were discussed in monthly ADE boards, where team members jointly reviewed medication regimens and proposed optimizations to reduce identified risks. The intervention also featured quarterly case conferences and regular audits using Plan-Do-Check-Act (PDCA) cycles, ensuring continuous quality improvement.

OAV was offered locally to home-care and nursing-home providers, with voluntary adoption permitting facilities to integrate the training and screening protocols into their routine practice. The approach assumes that heightened staff awareness, systematic risk assessment, and consistent interdisciplinary collaboration can reduce the incidence of ADEs and ultimately lower rates of hospital admission. Following staff training, the OAV intervention was integrated into routine care for all eligible residents irrespective of whether they had just entered the facility or had been receiving care for some time. This makes our intervention more real-world rather than study-setting oriented, especially compared to interventions that limit the type of patients included. For a detailed overview of the intervention design and implementation, please see the published study protocol [7].

Recognizing the distinct organizational structures of nursing homes and ambulatory care services (home care), the OAV intervention was delivered through a standardized, core methodology that allowed for setting-specific adaptations. All participating facilities received the same foundational components listed above. However, each site was encouraged to integrate these elements into its routine workflows in ways that best fit its day-to-day operations.

### Study design and data

The intervention was tested in a non-randomized, controlled, multi-center cohort design. Both nursing homes and ambulatory nursing care providers were recruited for participation to reflect the dual-sector reality of the provision of care for elderly patients. The participation of nursing facilities, as well as of care recipients, was voluntary.

A total of 65 nursing facilities, mainly from north-east Germany and to a small exctend from west Germany (North Rhine-Westphalia) were included in the intervention group, of which 58 were nursing homes and 7 were ambulatory nursing care providers. The different facilities started the intervention at different points in time between July 2018 and February 2020. Participants (i.e., care receipients) were prospectively recruited. Inclusion criteria were being insured by one of the collaborating sickness funds (AOK Nord-Ost, VIACTIV or IKK Berlin-Brandenburg) and receiving care from one of the 65 participating providers. Participation was fully voluntary, consent could be revoked at any time. A total of 1,566 patients were recruited, of which 1,444 were nursing home residents and 122 cared for by ambulatory nursing care providers.

Data were provided by the cooperating sickness funds and cover at least 18 full months before the start of the intervention in the respective nursing facility. The minimum required follow-up period was four full months, the longest follow-up observed in the data was 30 months. Partly observed months with incomplete data on health, treatments, medications, and costs were considered a potential source of bias and therefore excluded from the analysis.

Patients that died during follow-up after satisfying the minimum 4-month follow up observation period were not excluded from the analyses. Mortality trends before and after the intervention are shown in Fig. 5 in Appendix and appeared very similar for both groups.

### Health-related outcomes

The aim of the intervention was to reduce the incidence of ADEs (primary outcome) and hospital admissions (secondary outcome) by reducing the prevalence of polypharmacy and the incidence of PIM prescriptions. The prevalence of polypharmacy and the number of PIMs were included as process parameters reflecting intermediate outcomes.

ADEs were identified following Stausberg and Hasford [8], who listed 505 diagnosis codes from the international classification of diseases version 10 (ICD-10) system and categorized them by the strength of their association with ADEs. The strongest category A includes diagnoses which are considered as caused by an ADE (e.g. D52.1: drug-induced folic acid deficiency anemia or M10.29: drug-induced gout, unspecified site), the weakest category E includes diagnoses which may be related to an ADE, but may have other causes as well (e.g. E86: volume deficiency or I61.5: intracerebral intraventricular hemorrhage). In order to achieve high precision, we included only diagnoses from categories A-C (examples for category C: D69.0: anaphylactoid purpura or K71.5: toxic liver disease with chronic active hepatitis), where an ADE is considered to be at least a very likely cause [8]. We restrict the analysis to hospital admissions because hospital admissions were reported with the exact admission dates, whereas data from the German ambulatory sector was only available at a quarterly level. We only considered diagnoses which were reported as the reason for admission to ensure that the (potential) ADE is the reason and not a result of the hospital stay [8, 9]. In addition, we argue that the admission diagnosis is less likely to be biased due to coding issues (i.e., financial incentives) because the main and secondary are most relevant for DRG grouping and associated reimbursement, while the admission diagnosis is not directly related to reimbursement.

Finally, PIMs were defined as the 83 drugs on the PRISCUS list, which are considered to be potentially harmful for an elderly population, and should therefore not be prescribed to patients aged 65 or older, especially because safer alternatives are available [10–12].

### Costs

The monthly average costs of healthcare and nursing were used to measure potential changes in the sickness funds’ expenditures for ambulatory care, inpatient care, medicines, nursing services (SGB XI), hospice and palliative care, rehabilitation, medical aids (these include therapeutic remedies (e.g. physiotherapy or speech therapy) and medical aids/assistive devices (e.g. wheelchairs or prosthetics)), transportation, domestic help, and domestic nursing attributable to the intervention. As per study agreement, participating sickness funds payed €9.22 for nursing, €5.95 for physician services, €5.53 for pharmacies and €10.00 for medication analysis per month and participant, resulting in €30.70 as direct (variable) intervention costs per month and participant. One-time enrollment costs as per study agreement were €71.40 for nursing, €59.50 for physician services, €53.55 for pharmacies and €120.00 for medication analysis, resulting in €304.45 per participant. Over the average study participation of 16.04 months, one-time costs were €18.97 per month and enrolled participant. Training and sensitization costs amounted to €25.27 per patient and month. Thus, in total monthly costs of the intervention were €74.94 (€30.70 (direct) + €18.97 (one-time, monthly average) + €25.27 (training, monthly average).

### Subgroup analyses

We ran the Difference-in-Differences estimation for the whole sample after propensity score matching as well as for the subgroup of patients that actually received a medication analysis in order to test whether the effect differed in that subgroup.

### Empirical strategy

The quasi-experimental, staggered study design with potential self-selection into the intervention required a careful econometric approach. We accounted for this by combining an iterative Propensity Score Matching (PSM) with a difference-in-differences-estimator (DiD). PSM has its strengths in a posteriori control group selection and is particularly designed to overcome potential selection biases. However, it has two major limitations. First, it cannot control for unobservable heterogeneity in the study population, and second, it relies only on pre-intervention information. To overcome these limitations, we analyzed the dataset obtained through PSM using a DiD, following [13] who recommend combining these two methods when selection into treatment and control groups may somehow depend on the outcome of interest.

#### Propensity score matching

The control group was selected retrospectively in an iterative *three-stage PSM* approach, namely on the geographical level, nursing facility level and the participant level:

On the *geographical level*, we only included individuals from nursing homes and outpatient nursing care providers from the same federal states in which the intervention took place. Here, we matched the proportion of the control groups federal state to the intervention groups proportion of the same state to account for potential unobserved confounders on the regional-level.

On the *nursing-home level*, we stratified the nursing facilities in the intervention group by size (matching variables: number of beds for nursing homes, number of patients for mobile nursing care providers, both in intervals of 20), and for each nursing facility in the intervention group, six nursing facilities of similar size were randomly selected into the pool of potential controls. Inhabitants of the control facilities were eligible for matching if they were observed at least 12 months prior to the intervention uptake in the facility they were matched to. This step yielded an intervention to (potential) control group ratio of approximately 1:17, i.e., 26,593 individuals (21,597 in nursing homes and 4,996 receiving care from mobile nursing care providers) were available for the final PSM step.

**Fig. 1:**
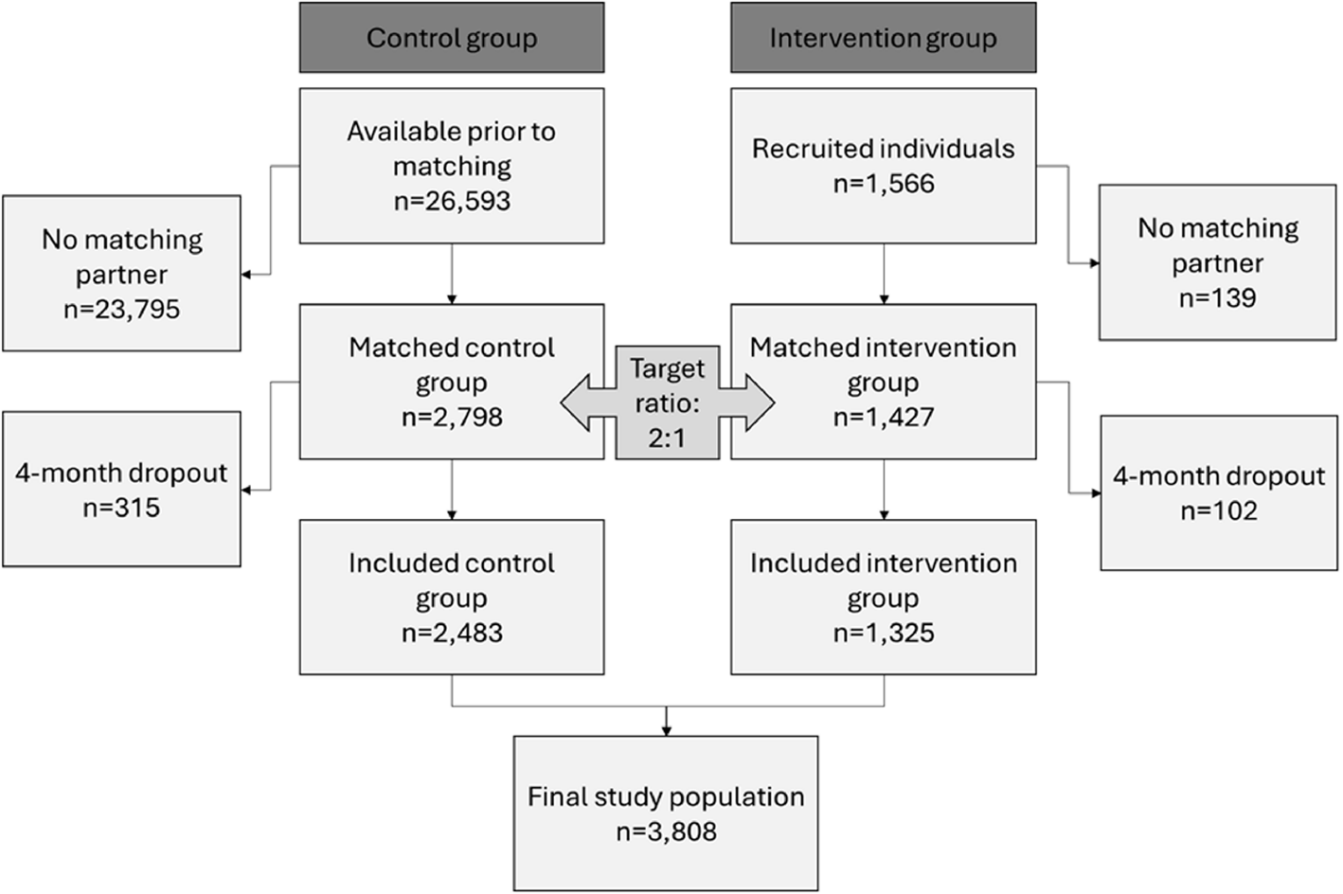
Patient recruitment and matching flow-chart

Finally, on the *individual level*, two controls per study participant were matched by a set of predictors of both study participation and primary outcome at the patient and the facility level [14] using the R-package *MatchIt* [15], where sampling from the data pool created in the first two steps took place without replacement [16].^1^ Following this step, 2,798 control and 1,427 intervention group individuals remained. Matched individuals of both groups were dropped if they were not observed for at least four months after the intervention started, because we assumed that the intervention may not have unfolded their full potential in a shorter observation time. Finally, 3,808 individuals were included in the study, of which 1,325 belonged to the intervention group and 2,483 to the control group (see Fig. 1).

To avoid problems arising from multicollinearity, variables with a variance inflation factor (VIF) > 10 were excluded from the PSM [17]. The balance between the intervention and control groups was assessed through the standardized mean differences (SMDs) [18].

The same starting point as observed for the respective participant was assigned to the matched controls as a placebo starting point to allow the comparison of trends relative to the starting points despite the staggered design. A caliper of 0.1 was used to restrict the potential for imbalances between intervention and control groups [19]. We repeated this final step for each starting point in time, beginning with the earliest intervention uptake in July 2018.

#### Difference-in-difference estimation

We used generalized linear models (GLM) with a DiD specification to identify the causal effect of the intervention on the primary and secondary outcomes. We denote

1 $$\begin{aligned} y_{ijt} = \mathcal {G} \left( a_t + \delta _1 d_{1,j} + \delta _2 \left( d_{1,j} \times d_{2,t} \right) + \sum _{k=1}^{K} \beta _k x_{k,it} + \sum _{l=1}^{L} \gamma _l z_{l,jt} + u_{ijt} \right), \end{aligned}$$

where $y_{ijt}$ is the observed outcome of individual $i$ in nursing facility $j$ at month $t$. $\mathcal {G}(\cdot )$ denotes a Poisson link function for the incidence of ADE-related hospital admissions, the incidence of total hospital admissions, and the number of PIMs (Lapane et al. 2011), and a logit link function for the presence of polypharmacy. The coefficient $\delta _2$ of the interaction term $d_{1,j} \times d_{2,t}$ is the DiD-estimator for the intervention, where dummy variable $d_{1,j}$ indicates that an individual is in the intervention group, and dummy variable $d_{2,t}$ indicates the post-intervention period. The control variables $x_{1,it}, \ldots, x_{K,it}$ account for potential confounders at the individual level, and the variables $z_{1,jt}, \ldots, z_{L,jt}$ control for potential facility-specific confounders (control variables are listed in Table 3). The term $a_t$ represents month-fixed effects which cover potential non-linear time effects, and $u_{ijt}$ denotes the error term. The coefficients were estimated by means of maximum likelihood estimation.

#### Cost effectiveness analyses

The cost-effectiveness of the intervention was assessed through the incremental cost-effectiveness-ratios (ICER) in comparison to the treatment-as-usual, i.e. by comparing it to the propensity-score-matched sample of individuals (control) who received the regular nursing and medical care. The ICER is computed as the average change in costs per average change in effects,

2 $$\begin{aligned} ICER = \frac{\overline{C}_{\text {intervention}} - \overline{C}_{\text {control}}}{\hat{\delta }_2}, \end{aligned}$$

where the average costs are taken from the routine data records and the change in the outcome is the estimated treatment effect $\delta _2$ from equation 2. To assess the precision of the estimated ICERs, we computed percentile-based confidence intervals based on 10,000 bootstrap replications [20, 21]. For this, we used the sample obtained from the PSM and obtained 10,000 bootstrap samples by drawing with replacement. We used these to repeat the complete estimation (regression analysis and computation of the ICER) 10,000 times and used the results for the statistical assessment of the ICER.

## Results

### Population characteristics and propensity score matching

Table 1 shows the means, standard deviations and the standardized mean differences (SMD) between intervention and control groups before and after matching. Before matching, groups differed (SMD > 0.1) regarding age, nursing home ratio, CarePlus enrollment, Charlson weighted score, number of different medicines taken, routine data costs and geography (federal state). After matching, all SMDs were < 0.1, indicating that intervention and control groups were sufficiently balanced [18]. The intended ratio of two controls per study participant could be achieved for almost all starting points in the staggered intervention design.

**Table 1:** Comparison of intervention and control groups before and after matching

	Intervention (*n*=1,325)		Control Before Matching (*n*=26,242)			Control After Matching (*n*=2,483)		
Variable	Mean	s.d.	Mean	s.d.	SMD	Mean	s.d.	SMD
Age	83.49	9.60	82.19	11.97	0.126	83.05	11.92	0.04
Male*	31.35%	0.46	31.0%	0.463	0.004	32.14%	0.47	0.02
In nursing homes*	91.96%	0.27	92.2%	0.269	0.357	91.65%	0.27	0.01
Enrolled in CarePlus*	19.49%	0.39	22.7%	0.419	0.522	19.41%	0.40	0.02
Health-related outcomes								
Charlson weighted score	4.98	2.84	4.374	2.947	0.223	4.98	2.81	0.00
ADE incidence (pre-int.)	1.66%	0.02	1.3%	0.113	0.021	1.80%	0.02	0.01
Hospital admissions (pre-int.)	9.50%	0.09	9.1%	0.288	0.043	10.11%	0.09	0.02
Medication								
Number of prescriptions	3.44	3.16	3.233	3.176	0.081	3.43	3.15	0.00
Number of different medicines	3.09	2.49	2.831	2.52	0.112	3.02	2.47	0.03
Number of prescribed PIMs	0.06	0.24	0.06	0.251	0.007	0.06	0.25	0.02
Polypharmacy*	25.24%	0.43	22.3%	0.416	0.075	23.20%	0.42	0.05
Cost (routine data) in €, preceding 12 months								
Ambulatory care	1359.51	2117.29	1146.959	2208.344	0.053	1423.38	2333.59	0.03
Ambulatory rehabilitation	3.87	67.56	3.979	102.412	0.003	0.49	12.45	0.07
Medicines	1900.14	2575.60	1668.309	3961.511	0.085	1770.20	2526.79	0.05
Remedy	754.34	1587.05	558.822	1337.648	0.154	739.67	1630.35	0.01
Medical aids	854.74	1312.25	716.417	1311.055	0.13	828.99	1396.97	0.02
Home nursing care	1091.13	3225.40	856.688	4571.872	0.067	1063.68	6974.77	0.01
Hospital care	6002.87	11601.42	4078.965	10657.822	0.171	5299.64	12366.29	0.06
Total for nursing care insurance	15527.01	7259.75	10933.315	8241.99	0.521	15640.28	7379.93	0.02
Rehabilitation	106.48	1020.03	86.013	930.927	0.012	109.43	1355.13	0.00
Palliative care	15.64	287.73	30.538	651.42	0.013	6.40	300.40	0.03
Transportation	923.13	1706.02	649.638	1598.467	0.179	845.80	1681.19	0.05
Total expenditure by SHI	13011.86	14764.20	9809.556	15279.181	0.17	12087.68	17284.52	0.06
Enrollment in DMPs								
Average number of DMPs	0.33	0.60	0.375	0.641	0.083	0.33	0.63	0.01
DMP asthma*	0.55%	0.10	0.8%	0.090	0.03	0.60%	0.10	0.01
DMP coronary heart disease*	10.06%	0.30	11.6%	0.320	0.05	10.61%	0.31	0.01
DMP breast cancer*	0.21%	0.00	0.2%	0.045	0.011	0.11%	0.00	0.03
DMP type 1 diabetes*	0.14%	0.00	0.1%	0.055	0.013	0.21%	0.00	0.02
DMP type 2 diabetes*	18.24%	0.38	20.5%	0.404	0.058	18.03%	0.38	0.01
DMP COPD*	2.98%	0.13	2.9%	0.176	0.051	3.29%	0.18	0.02
Federal state								
Lower Saxony*	0.07%	0.00	0.1%	0.031	0.016	0.04%	0.00	0.02
North Rhine-Westphalia*	4.16%	0.04	0.9%	0.094	0.257	2.97%	0.03	0.07
Berlin*	33.98%	0.22	37.4%	0.484	0.002	35.33%	0.23	0.02
Brandenburg*	37.73%	0.23	38.6%	0.487	0.009	37.59%	0.24	0.01
Mecklenburg-Western Pomerania*	24.06%	0.18	22.5%	0.419	0.004	24.01%	0.18	0.01

Standardized mean differences were obtained using the formulas in Yang and Dalton 2012 through the R-package stddiff. *: binary variables, s.d.: standard deviation $(\sqrt{\frac{1}{n} \sum _{i=1}^{n} (x_i - \bar{x})^2}$ for metric variables, $\sqrt{\bar{x}(1 - \bar{x})}$for binary variables)

*SMD* standardized mean difference (differences between the means in the intervention and control group divided by the average standard deviation), *DMP* Disease Management Program, *SHI* statutory health insurance, *ADE* adverse drug event

Within the first four months, 7.15% dropped out of the intervention group, and 11.26% dropped out of the control group, leaving us 1,325 patients in the intervention group and 2,483 patients in the matched control group (overall n = 3,808). Further investigating the composition of the matched sample showed that 8 of the 122 enrolled patients in outpatient care (6.56%) and 480 of the 1,444 enrolled patients in inpatient care (33.24%) received a medication analysis. The control group was taken from a larger number of facilities (Table 2).

**Table 2:** Summary of participant characteristics by group

	Intervention Group			Control Group		
	Home Care	Nursing Home	Total	Home Care	Nursing Home	Total
Number of Individuals	110	1215	1325	225	2258	2483
... with Medication Analysis	8	423	431	-	-	-
Number of Care Facilities	7	58	65	42	326	368

### Parallel trends assumption

The parallel trends assumption [22] was verified visually. Trends were smoothed by computing three-month-averages, and 95% confidence intervals (95%-CIs) of the smoothed trends were plotted to distinguish random variations from statistically significant trends. Parallel trends were compared for calendar months and for the months relative to the start of the intervention to account for the staggered intervention design.

**Fig. 2:**
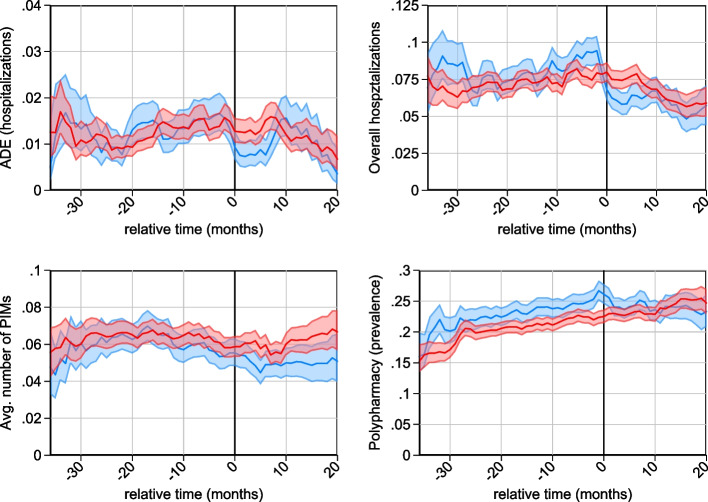
Parallel trends relative to treatment start. *Note*: Trends are smoothed to 3-month averages. Blue=intervention group; red=control group. Grey areas show 95% confidence intervals. Control group starts at a hypothetical start point (as the control group had no intervention), while the actual starting point was used for the intervention group. Month zero indicates the (hypothetical) start of the intervention

Figure 2 shows the trends for ADE, hospitalization, number of simultaneously prescribed PIMs and polypharmacy. Since the intervention started at different points in time in different nursing homes, we compared the trends between intervention and control groups after matching with the time relative to the starting points. The starting points in Fig. 2 are all set to zero, and the time scales are aligned such that negative time values indicate months before the intervention, and positive values indicate the time elapsed since the beginning of the intervention. The figure indicates similar trends for all four outcomes of interest. For the incidence of adverse drug events, total hospitalizations and the average number of PIMs prescribed, the trends were parallel, and the confidence intervals indicated no statistically significant differences in trend and level between the intervention group and the matched control group. The bottom right figure showed a statistically significant difference in the level between intervention and control groups for the prevalence of polypharmacy, but with a parallel trend before beginning of treatment.

**Fig. 3:**
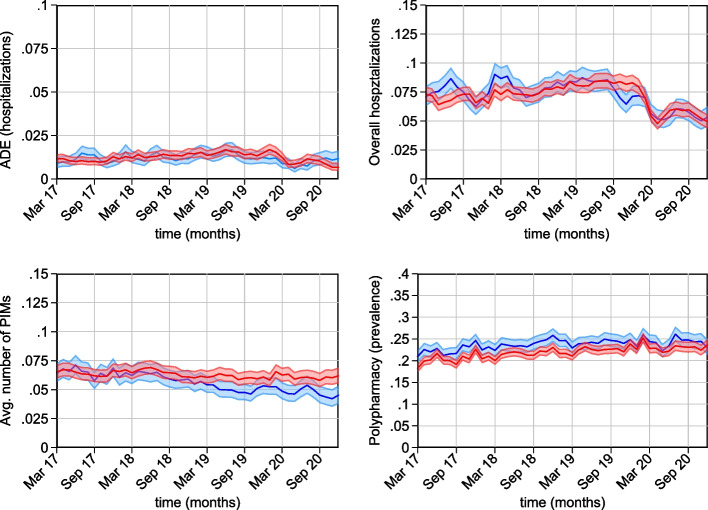
Parallel trends in actual months. *Note*: Comparison of trends for intervention and control groups with respect to observed dates. Blue=intervention group; red=control group. Outcomes are smoothed to 3 months averages; grey areas indicate 95% confidence intervals

Figure 3 presents the trends for intervention and control groups aligned by the observed date instead of the relative date and allow an assessment of potential confounders which may have occurred over the observation period. Since the intervention started between July 2018 and February 2020, the later starting points may have been affected by the onset of the COVID-19 pandemic in Germany in March 2020. The onset of the COVID-19 pandemic is reflected in the drop of ADE-related and total hospital admissions around March 2020, which occurred at the same time and in the same magnitude for both intervention and control groups. Since month-fixed effects were included to capture potential unobserved time-specific non-linear effects and the changes in March 2020 were similar in both groups, we assume that the potential for bias of the COVID-19 pandemic is accounted for by the regression design.

### Difference-in-differences analysis

Table 3 shows the IRRs for the count data outcomes ADE, hospital admissions, PIMs, and the OR for the presence of polypharmacy. The average treatment effect (ATE) is the interaction term of the indicator variable for being in the intervention group and the binary time variable indicating that the respective person-month was in the post-intervention period. The ATE for ADE, for example, suggests that the intervention decreased the incidence of ADE-related hospital admissions significantly by a factor of 0.725 (27.5%). The intervention effect for hospital admissions in general yielded a statistically significant IRR of 0.825, which suggests an approximately 17.5% lower admission rate.

Age and sex were significantly associated with hospital admissions, where the incidence and prevalence rates were higher among younger patients and among females. The quadratic specification allowed us to compute the analytical maximum of the expected incidence rates and risks with respect to the observed Charlson index. The turning points after which a higher Charlson index would be associated with a decreasing incidence or risk derived from the results are 13.2 for ADE and 12.3 for hospitalization, which are both inside the range of observed Charlson scores (0 to 16).

The health-related variables provided somewhat mixed results. Living in a nursing home, as compared to receiving ambulatory nursing care (home care), was not significantly associated with any of the outcomes, whereas enrollment in a DMP was associated with fewer ADEs and fewer hospitalizations. The estimated associations of enrollment in CarePlus ever in life and the indicator for a still active enrollment suggested that patients who were ever enrolled exhibit higher incidence rates of ADE and hospitalization. Contrarily, current enrollment decreased the respective rates statistically significantly.

Finally, point estimates of the difference-in-difference estimator indicated a small and statistically insigificant reduction in monthly total sickness fund costs as a consequence of the intervention (Table 3).

**Table 3:** Difference-in-difference estimation results

	ADEs^1^	Hospital Admissions^1^	Number of PIMs^1^	Polypharmacy^2^	Monthly Total Cost^3^
Treatment Effect	0.725*** (0.592, 0.887)	0.825*** (0.719, 0.948)	0.872* (0.755, 1.006)	0.936 (0.859, 1.020)	−23.083 (−101.525, 55.358)
Intervention Group	1.052 (0.908, 1.219)	1.073* (0.993, 1.158)	0.980 (0.827, 1.161)	1.137*** (1.035, 1.247)	50.619 (−15.718, 116.956)
*Patient Level Controls*					
Male	1.007 (0.876, 1.157)	1.111** (1.022, 1.207)	0.816** (0.680, 0.980)	0.804*** (0.736, 0.879)	−5.294 (−84.129, 73.542)
Age	0.994 (0.987, 1.002)	0.985*** (0.980, 0.990)	0.973*** (0.967, 0.980)	0.987*** (0.982, 0.992)	−22.371*** (−29.612, −15.131)
Charlson Weighted Score	1.481*** (1.384, 1.586)	1.372*** (1.311, 1.435)	1.074* (0.990, 1.164)	1.376*** (1.319, 1.435)	200.725*** (166.885, 234.565)
Squared Charlson Weighted Score	0.983*** (0.978, 0.988)	0.988*** (0.985, 0.992)	0.997 (0.990, 1.003)	0.987*** (0.984, 0.990)	−3.859*** (−6.700, −1.018)
Care Level (Reference=0)					
...1	1.461 (0.704, 3.034)	1.088 (0.760, 1.559)	0.779 (0.398, 1.523)	0.908 (0.470, 1.754)	−1021.544*** (−1449.623, −593.465)
...2	1.048 (0.658, 1.670)	1.232 (0.917, 1.659)	1.023 (0.642, 1.631)	1.177 (0.804, 1.725)	−268.294* (−541.033, 4.445)
...3	1.171 (0.747, 1.835)	1.219 (0.927, 1.605)	0.971 (0.636, 1.484)	1.106 (0.762, 1.606)	175.868 (−106.821, 458.558)
...4	1.093 (0.699, 1.709)	1.107 (0.841, 1.458)	0.915 (0.593, 1.411)	1.007 (0.691, 1.467)	582.261*** (303.938, 860.584)
...5	1.013 (0.639, 1.608)	1.051 (0.791, 1.397)	1.206 (0.783, 1.855)	0.971 (0.664, 1.419)	894.335*** (605.227, 1183.444)
*Healthcare Context Controls*					
In Nursing Home	0.998 (0.773, 1.289)	0.954 (0.807, 1.130)	1.146 (0.870, 1.510)	1.143* (0.994, 1.314)	86.866 (−113.166, 286.898)
Enrolled in DMP	0.864** (0.764, 0.976)	0.920** (0.858, 0.987)	0.956 (0.808, 1.132)	1.273*** (1.158, 1.399)	−173.207*** (−242.117, −104.298)
Ever Enrolled in CarePlus	1.650*** (1.328, 2.050)	1.751*** (1.523, 2.011)	0.891 (0.741, 1.070)	0.931 (0.797, 1.070)	185.175* (−22.253, 392.602)
CarePlus^4^ Period	0.420*** (0.327, 0.538)	0.399*** (0.341, 0.468)	1.342 (0.938, 1.919)	1.204* (0.997, 1.456)	−205.772*** (−388.867, −22.678)

Standard errors were clustered at the nursing home level. Time fixed effects were included in all analyses

***p< 0.01; **p< 0.05; *p< 0.1

^1^Coefficients presented as incidence-risk-ratios

^2^Coefficients presented as odds ratios

^3^Coefficients presented as differences in Euros

^4^We included an indicator variable for “enrolled in CarePlus” in our difference-in-differences model to account for baseline differences among facilities that had previously tested key OAV components from 2011 to 2017 under the pilot project known as “CarePlus”. Further control variables additional to the ones explicitly listed in Table 3 include month-fixed effects and state fixed effects

### Incremental cost of the intervention

During follow-up, the average monthly total sickness fund costs (=nursing and healthcare) were 21.84 Euro lower in the intervention group compared to the control group after PSM. This point estimate is remarkably close to the change in total sickness fund costs as identified by the difference-in-difference estimation in Table 3, where it was 23.08 Euro. Table 4 gives an overview of cost calculations for the intervention. Including the intervention-specific costs yielded incremental costs of 53.09 Euro per patient and month, but we consider this as a worst-case scenario because the compensations paid to the healthcare and nursing providers, as well as to the pharmacies, could be expected to be shared by more patients in case of an upscale and implementation into standard care.

**Table 4:** Calculation of incremental cost of the intervention. Incremental costs in bold font

Type of Cost	Cost in Euro per Patient-Month
Average cost difference in routine data (intervention - control)	EUR 21.84
*Implementation/intervention cost*	
Training and sensitization	EUR 25.27
Enrollment costs	EUR 18.97
Monthly compensation	EUR 30.70
Total implementation/intervention cost	EUR 74.94
Incremental cost of the intervention	**EUR 53.09**

Results for the subgroup of patients with medication analysis indicated that the intervention tended to be even more effective in this subsample of patients (Table 5 in Appendix).

### Cost-effectiveness of the intervention

We performed cost-effectiveness evaluation for ADEs (primary outcome) and hospitalizations (secondary outcome) based on bootstrapping with 10,000 repetitions. For the additional outcomes PIMs and polypharmacy, we provide ICER estimates without bootstrapped confidence intervals due to computational restrictions.

The intervention effects on ADE and hospitalizations were determined by computing the marginal effects. In addition to the reported incidence-risk-ratios presented in Table 3, the marginal effects indicate the expected change in the outcome per 100 patient-months.

**Fig. 4:**
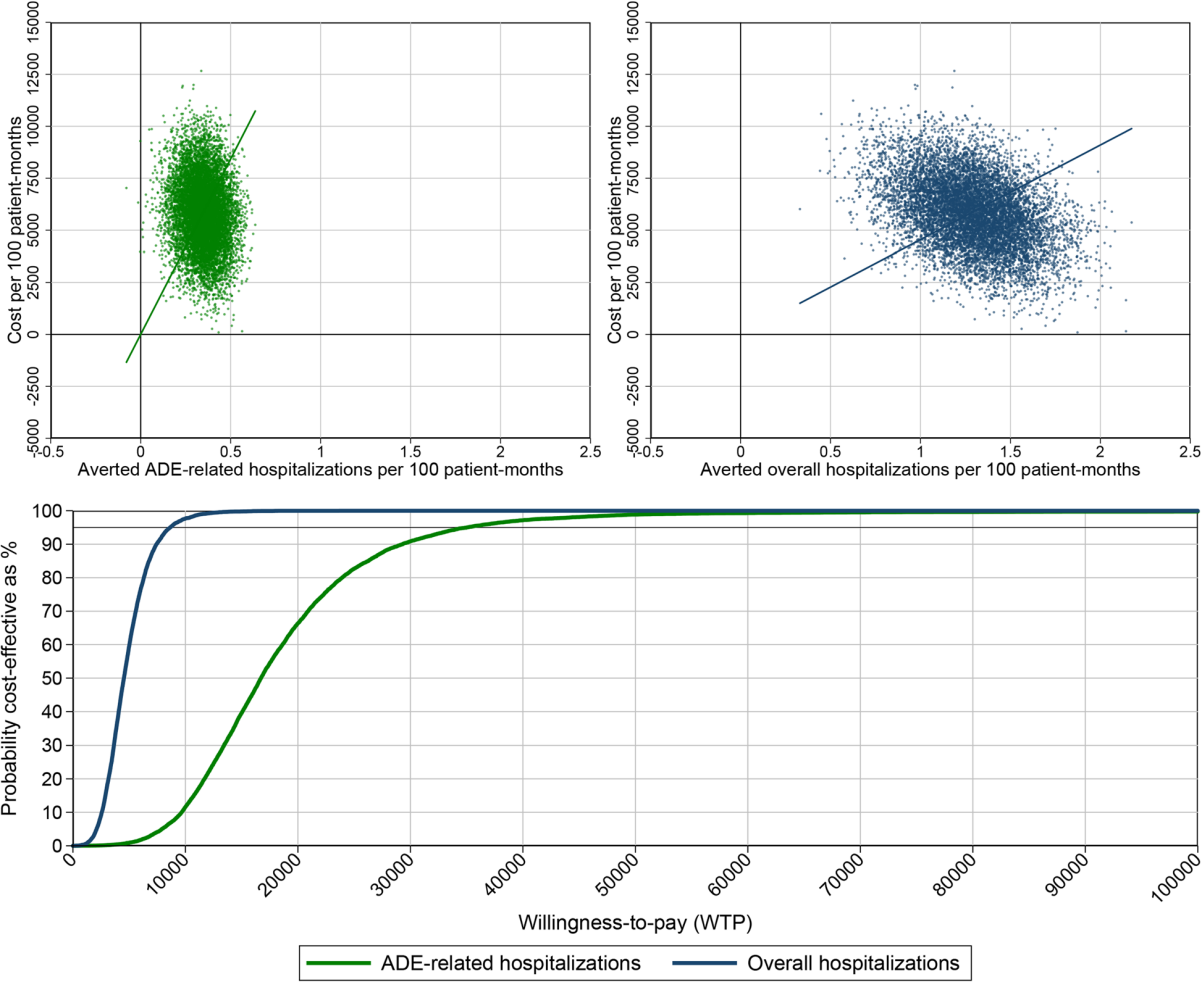
Averted ADEs. *Note:* Cost-effectiveness-planes for averted ADEs compared to total cost (top left) and to monthly cost only (top right) and corresponding cost-effectiveness-acceptability-curves (bottom). All estimated are controlled for patient level effects, nursing facility level effects and time fixed effects

**Fig. 5:**
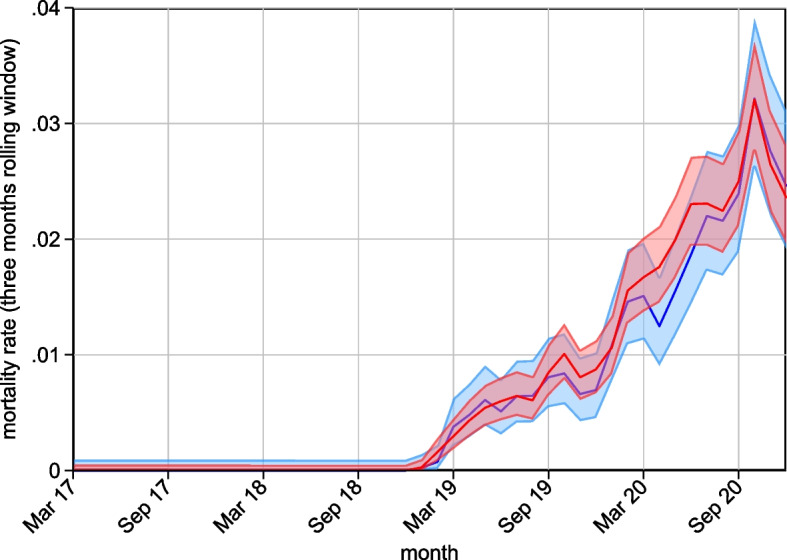
Mortality trends for matched intervention and control group

The marginal effects were 0.35 averted UAEs and 1.27 averted hospitalizations per 100 patient-months. The incremental cost per 100 patient-months were €5309.38. The ICER for averted ADE-related hospitalization is €15,169.66 per averted ADE, the ICER for overall averted hospitalizations is €4,180.61 per averted hospital admission (Fig. 4).

### Accuracy and statistical inference

The bootstrapped results are presented in Fig. 4. The scatterplots illustrate the results for ADE and hospitalization in the cost-effectiveness-plane, where the top left panel shows the results for averted ADEs and the top right panel shoes the results for averted all-cause hospitalizations. Almost all bootstrapped estimates with the exception of a few outliers were the top-right field, indicating that the intervention can reduce the incidences of ADEs and hospitalizations, but at higher costs compared to standard care. The CEACs in the bottom panel of Fig. 4 plot the probability of cost-effectiveness on the y-axis against a hypothetical willingness to pay (WTP) on the x-axis [23]: The intervention is cost-effective with zero probability up to a WTP of approximately 4,000 Euro per averted ADE, and is cost-effective with at least 95% probability for a WTP exceeding 35,000 Euro per averted ADE.

Because of the stronger effects for overall hospitalizations, the CEAC for averted hospitalizations is shifted to the left in Fig. 4 and indicates higher probability of cost-effectiveness for any WTPs below 60,000 Euros per averted hospitalization. The CEAC indicates that the intervention is cost-effective with at least 95% probability for a WTP exceeding 8,300 Euro per averted hospitalization.

## Discussion

This paper provided a practical example of a health economic evaluation, where the challenges involved by the quasi-experimental multi-center study design with staggered treatment uptakes and voluntary participation were overcome through an iterative PSM followed by a DiD estimation. The evaluation of this complex intervention, where nursing staff was sensitized for potential risks and signs of ADEs, and enrolled patients’ medication plans were reviewed if deemed necessary, was taken as an example. We specifically developed an advanced iterative PSM strategy to take the clustering of patients, the geographical heterogeneity and the chronology of intervention uptakes across different nursing providers into account. The approach ensured that treatment and intervention groups were sufficiently balanced for a reliable impact evaluation. We then evaluated the intervention using a DiD approach to estimate the ATE, thereby controlling for observed covariates and unobserved heterogeneity. The empirical results indicate that the intervention reduced the incidence of ADE-related hospital admissions by 27.5 percent, and the incidence of total hospital admissions by 17.5 percent. The ICERs were 15,169.66 per averted ADE-related hospitalization and 4,180,61 per averted all-cause-hospitalization. These numbers mark the upper bounds of the ICERs to be expected in a real-world implementation, because training costs could be borne by more patients over longer periods of time than in this study.

The present study design required a new and unique approach to allow for a proper impact evaluation. Existing papers which combined PSM and DiD estimation [24] or used propensity score weights in DiD estimation [25] did not cover the staggered design encountered in this study. Furthermore, recent papers on the analysis of staggered designs with DiD analyses suggested, for example, to use the individuals from the intervention group as controls in periods in which they were not treated [26–28]. This approach was not applicable here for two reasons: First, this would require the limited treatment assumption and the random treatment assignment assumption to hold true [29]. However, it was impossible to randomize treatment assignment or time of uptake. In addition, the intervention was assumed to effect medication plans and health outcomes for more than the month of intervention uptake, whereas the limited treatment assumption would require that treatments only effect individuals while performed, but not in periods before or after the treatment. Second, sufficient overlaps of observation periods for individuals from different entities were not ensured at all times, since not all participants and nursing providers were observed over the whole study period. Further, instead of using the intervention group as control in times where no treatment change occurred, a separate, never-treated control group existed. We therefore developed and applied a multi-stage PSM in order to select a suitable control group from the routine data pool, and additionally applied a DiD estimation strategy, where we refined the specification of Sun und Abraham [30] by accounting for time-fixed effects in addition to the distinction between pre- and post-intervention periods.

Our results diverge from the previous literature on interventions aiming to improve health outcomes by means of changes in medication plans. While we found that the intervention decreased ADE-related and overall hospitalizations, other studies found no effect of comparable interventions on overall hospital admissions [31–34] or ADE-related hospital admissions [35]. The reduction of ADE-related hospital admissions in our study was similar to the effect of an electronic warning system for potentially harmful medications [36]. Similar to Junius-Walker [31], we found no statistically significant effect on the number of dispensed PIMs. Two studies investigating more holistic initiatives to improve quality of care in nursing homes, specifically designed to reduce avoidable hospitalizations, reported even stronger reductions of the incidence of hospitalizations [37, 38].

Although the intervention decreased the incidence of ADE-related and overall hospitalizations, no statistically significant reduction in polypharmacy was found. A potential explanation for not observing changes in polypharmacy as an intermediate outcome is that certain medications within a drug class may have been substituted. Even if certain medications were discontinued, the number of different drugs may have changed, but not necessarily fallen below the threshold of five simultaneously taken drugs in cases where polypharmacy was observed prior to the intervention. We tested whether the number of different prescribed active ingredients changed as a result of the intervention using our specified DiD model with the number of different prescriptions as outcome. We found a small but statistically insignificant decrease (IRR=0.976, p=0.149) in the number of different simultaneously prescribed drugs. Another possible explanation could be that increased awareness created by the intervention led to temporary discontinuation or dose reduction, neither of which would be detectable by the indicators used, but might explain the reduction in ADE-related and all-cause hospitalizations to some extent.

The intervention was associated with a small reduction in the average cost of routine care (Table 4), however, preventing ADE-related and overall hospital admissions through the intervention involved significantly higher costs when taking the direct costs of the intervention into account. Therefore, in practice, willingness-to-pay for averted hospitalizations needs to be considered to assess whether the intervention is cost-effective.

### Limitations

The present study has several limitations. First, we relied on routine data for evaluating the effectiveness of the intervention under study. The data utilized, collected primarily for administrative and billing purposes, lack clinical information such as symptoms, laboratory, imaging results and patient-reported outcomes. In addition, the routine data coding may be insufficiently detailed in order to capture differences between patient with respect to severity of diseases. Thus, the data does not capture the full spectrum of a patient’s health status or the progression of their disease over time leading to unobserved factors that could potentially lead to inaccurate assessments of the intervention’s effectiveness. To overcome this limitation, we employed a combined approach of PSM and DiD analyses. PSM was used to ensure that the treatment and control groups are comparable in terms of observed characteristics, while DiD was instrumental in controlling for unobserved, time-invariant confounders. This dual approach enhances the robustness of our findings, addressing both the issues of non-randomized group selection and potential unobserved confounding variables.

Second, our method for identifying ADEs focused exclusively on ADE-related hospitalizations. Consequently, this approach overlooked effects on milder ADEs not requiring inpatient treatment. This exclusion likely led to an underestimation of the total incidence of ADEs in the study population. The implications of this limitation for the cost-effectiveness of the intervention under study are not entirely clear. It remains unclear whether the intervention similarly reduced ADEs that were treated in an ambulatory setting as it did for those necessitating hospitalization. There is also a possibility that more severe ADEs, which required hospitalization prior to the intervention, might have been reduced to less severe, now unobservable, ADEs manageable in an ambulatory setting. Furthermore, it is possible that incorporating certain ICD-10 codes based on the categorization by Stausberg and Hasford (2011), specifically those within categories A-C, might have led to the inclusion of diagnoses that do not genuinely represent ADEs. This inclusion could have artificially inflated the reported incidence of ADEs, given that some of these diagnoses might not be accurately classified as ADEs. Consequently, this casts uncertainty on the actual incidence of ADEs in our study.

Third, the direct intervention costs are subject to uncertainty, especially since the costs per person depend on the duration of participation in the intervention. Additionally, the training costs per intervention participant depend on both the size of the nursing home and the number of trained staff members. Since only a 12-month observation period was considered here, and the training had to be financed as a one-time investment in the training of the staff in each participating nursing home, the direct costs of the intervention per study participant may have been overestimated. Thus, the estimates of costs and ICER should be considered conservative.

Fourth, we cannot rule out that a change in mortality as a result of the intervention impacted on outcomes and costs, however, assessing the potential consequences is difficult. For example, reviewing medication plans and preventing side effects and ADEs may have prolonged life expectancy and increased the costs of routine care in the intervention group prolonging the life expectancy of the study participants who were already at the end of life, resulting in higher costs (compression hypothesis, delaying high costs shortly before death) [39]. Additionally, a lower mortality rate could have increased the likelihood of ADE-related or overall hospital admissions if the longer-living individuals in the intervention group had a higher probability of hospitalization not related to medications. If these individuals had died without the intervention, hospital admission would not have been possible. In this case, the observed effect of the intervention would have been underestimated. However, it is unknown to what extent the intervention actually influenced the mortality of the participants.

Fifth, selection bias both at the individual and the facility level may be present in our study. The intervention group consisted of individuals and nursing homes that actively agreed to participate in the study. On the facility level, participating nursing homes may be those that are especially engaged in improving patient outcomes and therefore represent above-average care providers regarding quality. On the individual level, participants may be more conscious regarding their own health than the average nursing home resident. Therefore, we likely studied a sample that is not representative of the general long-term care-dependent population of Germany, hence limiting the external validity of our findings. Therefore, further research to where comparable interventions are implemented to a broader set of nursing facilities is necessary.

Sixth, we used the average cost difference instead of that obtained from the Difference-in-Differences estimator because of the long runtime of the estimation program. We argue that the difference in the two estimates, 23.08 Euros compared to 21.84 Euros, is small enough to assume that the cost difference is precise enough to warrant the reduction in processing time at the potential cost of a small imprecision in the estimated cost difference.

Seventh, our sample includes patients from nursing homes as well as outpatient nursing facilities, reflecting slightly different populations. Although the intervention was carried out in a comparable pattern across both types of facilities, one may argue that the treatment effects would have been different had the intervention been carried out only in one type of setting. We tried to account for this by balancing control group and intervention group regarding outpatient vs. inpatient setting, and further controlling for nursing home status in our in DiD models. On the contrary, others may argue that incorporating outpatient nursing facilities may improve representitiveness of our analysis.

Finally, the SARS-CoV-2 pandemic and the associated restrictions [40] may have influenced decisions regarding ADE-related and overall hospital admissions. Particularly during the first wave of the SARS-CoV-2 pandemic in Germany from March 1 to June 30, 2020 [41], the incidence of ADE-related and overall hospital admissions decreased markedly. In this period, elective care tended to be postponed and public life was widely shut down to prevent the healthcare system from collapsing. During that time, fear of infections and of spreading the virus across healthcare and nursing facilities led to forgone elective treatments, isolation of residents in nursing homes and avoidance of hospital admissions. Since we observed almost identical drops in the intervention and control groups, we argue that the pandemic equally affected both groups, particularly due to the temporal and geographical matching procedure. In addition, the period-fixed effects allowed us to control for discontinuities in individual time periods, such that distortions due to the SARS-CoV-2 pandemic are rather unlikely.

## Conclusion

The study presented a unique approach to overcome challenges arising from a non-randomized, quasi-experimental multi-center trial with differences in treatment timing and observation periods. Developing an advanced PSM approach to prepare our DiD analysis, allowed us to estimate the effect of the intervention on various outcomes, which can be interpreted as a causal effect of the intervention. The intervention was effective in preventing ADEs and hospital admission, although to the detriment of increased costs when accounting for the extra spending for the intervention. Depending on willingness-to-pay for averted ADE-related and all-cause hospital admissions beyond the potential avoidance of costs for inpatient care, the intervention may still be cost-effective.

## Data Availability

The data that support the findings of this study are available from AOK Nordost but restrictions apply to the availability of these data, which were used under license for the current study, and so are not publicly available. Data are however available from the authors upon reasonable request and with permission of AOK Nordost.

## Appendix

**Table 5:** Difference-in-difference estimation results for patients with medication analysis

	ADEs^1^	Hospital Admissions^1^	Number of PIMs^1^	Polypharmacy^2^
Intervention Effect	0.689*** (0.592, 0.887)	0.834*** (0.719, 0.948)	0.685* (0.755, 1.006)	0.833 (0.859, 1.020)
Intervention Group	0.938 (0.908, 1.219)	1.121* (0.993, 1.158)	1.335 (0.827, 1.161)	1.551*** (1.035, 1.247)
*Patient Level Controls*				
Male	1.007 (0.876, 1.157)	1.111** (1.022, 1.207)	0.891** (0.680, 0.980)	0.945*** (0.736, 0.879)
Age	0.994 (0.987, 1.002)	0.985*** (0.980, 0.990)	0.985*** (0.967, 0.980)	0.986*** (0.982, 0.992)
Charlson Index	1.637*** (1.384, 1.586)	1.464*** (1.311, 1.435)	1.324* (0.990, 1.164)	1.384*** (1.319, 1.435)
Squared Charlson Index	0.975*** (0.978, 0.988)	0.982*** (0.985, 0.992)	0.985 (0.990, 1.003)	0.986*** (0.984, 0.990)
Care Level (Reference=0)				
...1	3.586 (0.704, 3.034)	0.758 (0.760, 1.559)	0.165 (0.398, 1.523)	0.119 (0.470, 1.754)
...2	1.004 (0.658, 1.670)	0.984 (0.917, 1.659)	0.810 (0.642, 1.631)	1.177 (0.804, 1.725)
...3	1.235 (0.747, 1.835)	1.169 (0.927, 1.605)	1.000 (0.636, 1.484)	1.038 (0.762, 1.606)
...4	1.059 (0.699, 1.709)	1.011 (0.841, 1.458)	1.138 (0.593, 1.411)	1.104 (0.691, 1.467)
...5	1.070 (0.639, 1.608)	0.957 (0.791, 1.397)	1.697 (0.783, 1.855)	0.969 (0.664, 1.419)
*Healthcare and Nursing Context Controls*				
In Nursing Home	0.913 (0.773, 1.289)	0.913 (0.807, 1.130)	1.857 (0.870, 1.510)	1.433* (0.994, 1.314)
Enrolled in DMP	0.944** (0.764, 0.976)	1.024** (0.858, 0.987)	1.176 (0.808, 1.132)	1.303*** (1.158, 1.399)
Ever Enrolled in CarePlus	1.650*** (1.328, 2.050)	1.650*** (1.523, 2.011)	1.342 (0.741, 1.070)	1.204* (0.797, 1.070)
CarePlus Period	0.420*** (0.327, 0.538)	0.399*** (0.341, 0.468)	0.942 (0.938, 1.919)	1.204* (0.997, 1.456)

Standard errors were clustered at the nursing home level. Time fixed effects were included in all analysis

****p*< 0.01; ***p*<0.05; **p*< 0.1

^1^Coefficients presented as incidence-risk-ratios

^2^Coefficients presented as odds ratios

## Abbreviations

ADE: Adverse drug event
DiD: Difference-in-differences
ICER: Incremental cost-effectiveness ratio
PSM: Propensity score matching
PIM: Potentially inadequate medication
ICD-10: International classification of diseases, 10th revision
DRG: Diagnosis-related group
SMD: Standardized mean difference
VIF: Variance inflation factor
GLM: Generalized linear model
IRR: Incidence-rate ratio
OR: Odds ratio
DMP: Disease management program
SHI: Statutory health insurance
CEAC: Cost-effectiveness acceptability curve
WTP: Willingness-to-pay

## Footnotes

1 The matching variables included federal state (as a factor), age, nursing home residency, participation in the CarePlus program (a previously tested program that utilized key OAV components from 2011 to 2017 under the pilot project known as “CarePlus”), gender, participation in disease management programs (DMP) as an indicator of willingness to seek help, the weighted comorbidity score (wscore), and various healthcare costs over the past 12 months, including outpatient, hospital, nursing care, hospice, specialized palliative care (SAPV), ambulatory rehabilitation, home assistance, home nursing care, medical aids, therapeutic treatments, pharmacy, rehabilitation, and transportation costs. Additionally, the matching incorporated care level (as a factor), the number of diagnoses, and comorbidities based on the Charlson categories, including myocardial infarction (AMI), congestive heart failure (CHF), peripheral vascular disease (PVD), cerebrovascular disease (CEVD), dementia, chronic obstructive pulmonary disease (COPD), peptic ulcer disease (PUD), mild liver disease (MLD), diabetes (DIAB) with and without complications (DIABWC), renal disease (REND), hemiplegia/paraplegia (HP), metastatic cancer (METACANC), moderate to severe liver disease (MSLD), and AIDS.
